# Role of Interleukin-10 in Acute Brain Injuries

**DOI:** 10.3389/fneur.2017.00244

**Published:** 2017-06-12

**Authors:** Joshua M. Garcia, Stephanie A. Stillings, Jenna L. Leclerc, Harrison Phillips, Nancy J. Edwards, Steven A. Robicsek, Brian L. Hoh, Spiros Blackburn, Sylvain Doré

**Affiliations:** ^1^College of Medicine, University of Florida, Gainesville, FL, United States; ^2^Department of Anesthesiology, College of Medicine, Center for Translational Research in Neurodegenerative Disease, University of Florida, Gainesville, FL, United States; ^3^Department of Neuroscience, College of Medicine, University of Florida, Gainesville, FL, United States; ^4^Department of Anesthesiology, University of Florida, Gainesville, FL, United States; ^5^Department of Neurology, University of California, San Francisco, CA, United States; ^6^Department of Neurosurgery, University of California, San Francisco, CA, United States; ^7^Department of Neurosurgery, University of Florida, Gainesville, FL, United States; ^8^Department of Neuroscience, University of Florida, Gainesville, FL, United States; ^9^Department of Neurosurgery, University of Texas, Houston, TX, United States; ^10^Department of Neurology, Center for Translational Research in Neurodegenerative Disease, McKnight Brain Institute, University of Florida, Gainesville, FL, United States; ^11^Department of Psychology, Center for Translational Research in Neurodegenerative Disease, McKnight Brain Institute, University of Florida, Gainesville, FL, United States; ^12^Department of Psychiatry, Center for Translational Research in Neurodegenerative Disease, McKnight Brain Institute, University of Florida, Gainesville, FL, United States; ^13^Department of Pharmaceutics, Center for Translational Research in Neurodegenerative Disease, McKnight Brain Institute, University of Florida, Gainesville, FL, United States

**Keywords:** concussion, endothelium, intracerebral hemorrhage, ischemia, stroke, subarachnoid hemorrhage, traumatic brain injury, vasculature

## Abstract

Interleukin-10 (IL-10) is an important anti-inflammatory cytokine expressed in response to brain injury, where it facilitates the resolution of inflammatory cascades, which if prolonged causes secondary brain damage. Here, we comprehensively review the current knowledge regarding the role of IL-10 in modulating outcomes following acute brain injury, including traumatic brain injury (TBI) and the various stroke subtypes. The vascular endothelium is closely tied to the pathophysiology of these neurological disorders and research has demonstrated clear vascular endothelial protective properties for IL-10. *In vitro* and *in vivo* models of ischemic stroke have convincingly directly and indirectly shown IL-10-mediated neuroprotection; although clinically, the role of IL-10 in predicting risk and outcomes is less clear. Comparatively, conclusive studies investigating the contribution of IL-10 in subarachnoid hemorrhage are lacking. Weak indirect evidence supporting the protective role of IL-10 in preclinical models of intracerebral hemorrhage exists; however, in the limited number of clinical studies, higher IL-10 levels seen post-ictus have been associated with worse outcomes. Similarly, preclinical TBI models have suggested a neuroprotective role for IL-10; although, controversy exists among the several clinical studies. In summary, while IL-10 is consistently elevated following acute brain injury, the effect of IL-10 appears to be pathology dependent, and preclinical and clinical studies often paradoxically yield opposite results. The pronounced and potent effects of IL-10 in the resolution of inflammation and inconsistency in the literature regarding the contribution of IL-10 in the setting of acute brain injury warrant further rigorously controlled and targeted investigation.

## Introduction

Stroke and traumatic brain injury (TBI) are devastating acute neurological disorders that can result in high mortality rates or long-lasting disability. Approximately 87% of strokes are ischemic and 13% are hemorrhagic, with 10 and 3% of the latter representing intracerebral hemorrhage (ICH) and subarachnoid hemorrhage (SAH), respectively (1). Stroke is the fourth most common cause of death in the United States, and ischemic stroke (IS) in particular is the seventh most frequent emergency department presentation (2, 3). TBI and concussions have over twice the incidence of all strokes combined (4), with more than three million people in the United States alone living with long-term disability as a result of TBI (5). Collectively, stroke and TBI have very few treatments, and despite advances in clinical management of these disorders, they are still associated with significant disability and mortality (6, 7).

Inflammation plays a central role in the pathophysiology of stroke and TBI and can have both protective and harmful effects on brain tissue (8–15). Although there are some distinct differences in the inflammatory cascades following the various types of acute brain injury, there are also numerous commonalities. Acute neuroinflammation is characterized by the activation of resident central nervous system (CNS) immune surveillance glial cells that release cytokines, chemokines, and other immunologic mediators, which facilitate the recruitment of peripheral cells such as monocytes, neutrophils, and lymphocytes (8, 9, 12, 15). Collectively, this initial response is helpful in the clearance of toxic entities and the restoration and repair of damaged tissue. However, during the resolution phase, with an uncontrolled and prolonged inflammatory response, secondary damage results from overactivation of this inflammatory surge and release of additional factors that led to breakdown of the blood–brain barrier (BBB), cerebral edema, cerebral hypertension, and ischemia.

Interleukin-10 is generally known as an anti-inflammatory cytokine that exerts a plethora of immunomodulatory functions during an inflammatory response and is particularly important during the resolution phase. Expression of IL-10 in the brain increases with CNS pathology, promoting neuronal and glial cell survival, and dampening of inflammatory responses *via* a number of signaling pathways (16). IL-10 was originally described as cytokine synthesis inhibitory factor and in addition to attenuating the synthesis of proinflammatory cytokines, IL-10 also limits inflammation by reducing cytokine receptor expression and inhibiting receptor activation (16). Furthermore, IL-10 has potent and diverse effects on essentially all hematopoetic cells that infiltrate the brain following injury. For example, IL-10 reduces the activation and effector functions of T cells, monocytes, and macrophages, ultimately ending the inflammatory response to injury (17). The structure, function, and regulation of IL-10 have been extensively reviewed elsewhere, including a review of IL-10 in the brain (16–20), although not in the context of the various forms of acute brain injury. Please refer to the aforementioned reviews for additional details, including the potential cellular sources, target cells, signal transduction, and mode of action of IL-10.

Given the intriguing multifactorial role of IL-10 in the resolution of inflammatory cascades that are important for promoting neurologic recovery from acute brain injury, here we present a comprehensive literature review of preclinical and clinical studies in this area. We focus on the contribution of IL-10 in modulating various important parameters and pathophysiologic processes important for IS, SAH, ICH, and TBI outcomes, and whether IL-10 has therapeutic or biomarker potential. A better understanding of the many functions of IL-10 in the brain after injury, particularly in the resolution phase of inflammatory processes, will promote our knowledge of the pathophysiology of these debilitating disorders and guide future development of novel therapeutic approaches.

## Vascular Endothelium, Remodeling, and Dysfunction

The neurovascular unit, including the vascular endothelium, has become a therapeutic target of interest in the various types of acute brain injury (14, 21–24). IL-10 has an assortment of functions acting at the vascular and endothelial level, such as modulating vascular remodeling, reducing leukocyte adhesion and extravasation, mitigating leukocyte–endothelial interactions that facilitate coagulation, promoting vasodilatation *via* increased production of nitric oxide, and direct protection of the endothelium from oxidative stress *via* the downregulation of harmful reactive oxygen species (ROS)-producing enzymes, and/or the upregulation of antioxidant pathways.

### IL-10 in Preclinical Studies

Interleukin-10 deficiency results in a spectrum of problems with the vasculature, including everything from vascular and endothelial damage from increased oxidative stress and inflammation to deleterious remodeling and an inability of the vasculature to respond to physiological demands such as the requirement for vasodilation. IL-10 inhibits Nox1, a subunit of NADPH oxidase, which plays a role in degenerative vascular remodeling by generating ROS, causing oxidative stress (25). IL-10 knockout (IL-10^−/−^) mice have higher levels of Nox1, Nox2, and p22^phox^ (two additional NADPH oxidase components) and superoxide production and display decreased aortic medial thickness, a loss of smooth muscle cells, and increased vascular collagen deposition, indicating harmful vascular remodeling with IL-10 deficiency (25, 26). Administration of a superoxide scavenger in IL-10^−/−^ mice prevented vascular remodeling, suggesting the oxidative stress-dependent mechanism (ROS formation by NADPH oxidase) of injurious vascular remodeling with IL-10 deficiency (26). Additionally, IL-10 deficiency led to increased levels of matrix metalloproteinase-9 (MMP-9) in aortic smooth muscle cells, IL-6 in aortas, and the vasoconstrictor endothelin-1 in plasma (25).

Interleukin-10 attenuates endothelial dysfunction and vasoconstriction mediated by ROS, endothelin-1 (27), angiotensin II (28), and by ischemia-reperfusion injury (29), among other mediators, and these protective mechanisms may become more important with age. Old, but not young, IL-10^−/−^ mice have diminished vasodilatory responses to acetylcholine, while the nitroprusside response is intact, suggesting endothelial rather than vascular smooth muscle dysfunction (26). Additionally, viral transduction of canine basilar arteries with IL-10 increases vasodilatory responses and reduces levels of ICAM-1 and VCAM-1, further supporting the protective role of IL-10 on the endothelium (29). IL-10 has also been shown to act *via* the AKT pathway to decrease TNFα-directed ceramide synthesis, resulting in lower levels of ROS and ICAM-1 (30). In aortic rings from IL-10^−/−^ mice, TNFα reduces endothelial nitric oxide synthase expression and vasodilatory ability, and IL-10 administration restores function, providing a protective effect (31). A similar experiment showed that murine aortic rings treated with angiotensin II showed impaired relaxation that was reversible with IL-10 administration (32). High cyclooxygenase-2 activity also plays a role in these stiffer vessels seen in IL-10^−/−^ mice, ultimately resulting in decreased vascular relaxation, impaired cardiac function, and a larger heart size (33).

The source of endothelial protective IL-10 may come from CD4^+^CD25^+^ regulatory T cells (Tregs) (34), as well as the various other types of IL-10-producing cells, including B cells (see the “IS” section below). For example, hypertensive IL-10^−/−^ mice transduced with hypertensive wildtype (WT) Tregs have a better vasodilatory response to acetylcholine and lower levels of NADPH oxidase, whereas hypertensive IL-10^−/−^ Tregs does not confer protection on hypertensive WT mice (34). In middle-aged spontaneously hypertensive rats that display features similar to early-stage human cerebral small vessel disease, there is a decreased level of IL-10 in the cerebrospinal fluid and other immune changes (35).

Of interest, inflammation mediated by toxic bacterial agents, such as lipopolysaccharide (LPS), is more severe in IL-10^−/−^ mice (36). Surprisingly, cerebral bacteremia may result in so much endothelial damage in the absence of IL-10 as to cause ICH and death shortly following peak bacterial loads (37). These findings are associated with increases in the FAS/FAS-ligand apoptotic pathway, which IL-10 reverses (37). However, the effects of IL-10 seem to be specific to the noxious stimuli generating its production. For example, IL-10 produced in response to both LPS and *Borrelia burgdorferi* reduces lymphocyte endothelial migration and blunts endothelial production of chemokines; however, IL-10 produced in response to IL-1β and TNFα does not show such effects (38).

### Summary of Evidence Describing the Role of IL-10 in Protecting the Vasculature

**Figure 1:**
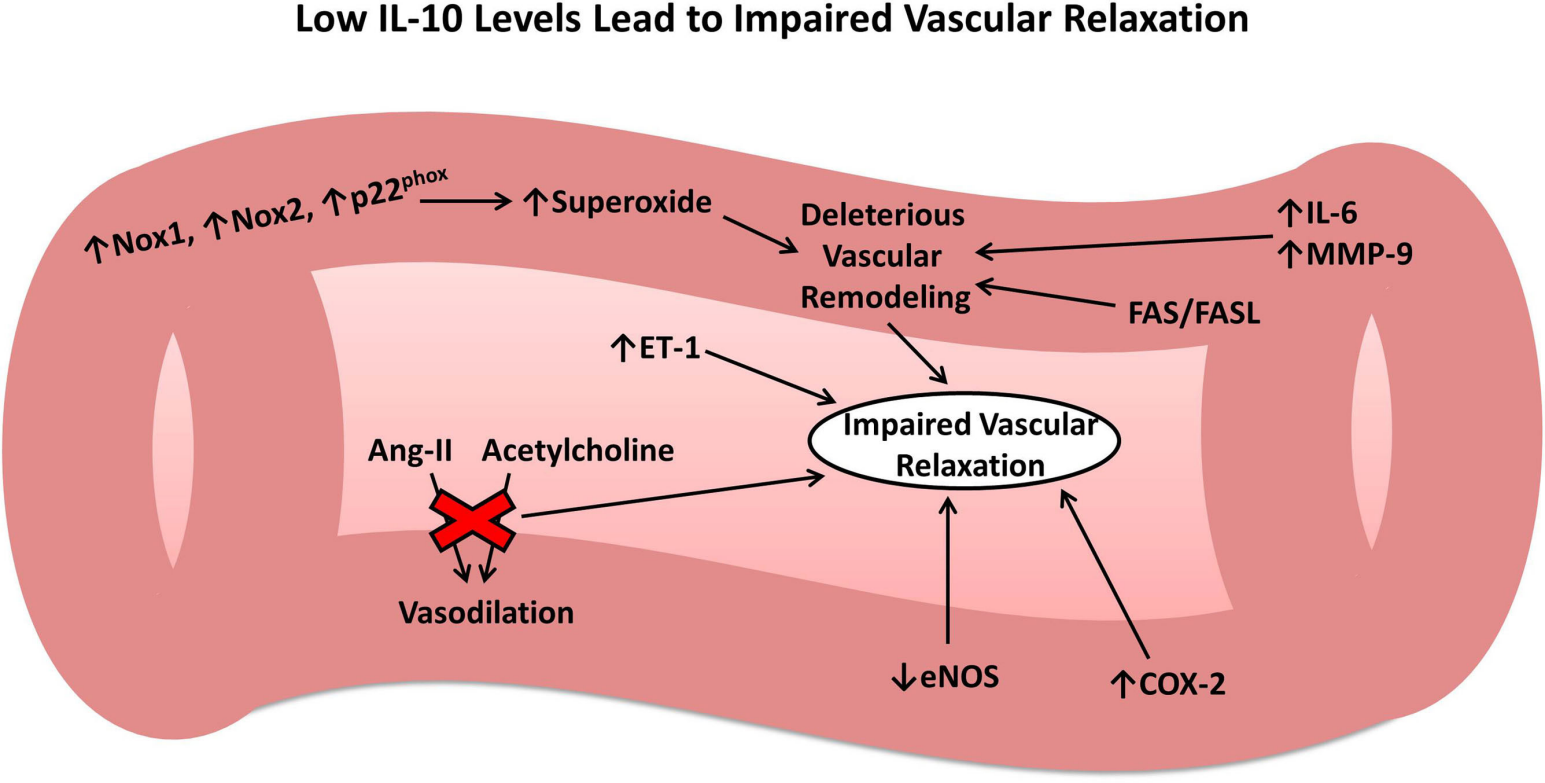
Role of interleukin-10 (IL-10) in vascular remodeling and dysfunction. Low or absent IL-10 results in numerous changes to the vasculature leading to harmful vascular remodeling and impaired vascular relaxation in response to important physiologic mediators.

Given the intimate tie between the vascular endothelium and pathophysiology of all forms of acute brain injury, it is important to consider the effects of IL-10 on the vasculature. The above studies have shown that following injury, the inflammatory system works on many different levels to cause endothelial cell damage and vascular dysfunction. IL-10 appears to play a central and multifaceted role in attenuating these effects and facilitating the resolution phase of the inflammatory system. In contrast, low or absent IL-10 leads to several changes in gene expression that ultimately results in deleterious vascular remodeling and impaired vascular relaxation in response to physiologic mediators (Figure 1), outcomes that would exacerbate secondary brain damage following acute injury. In summary, IL-10 plays a crucial role in restoring vascular function following injury to the vasculature, similar to that which occurs after IS, SAH, ICH, and TBI.

## Ischemic Stroke

Ischemic stroke occurs when a local thrombus or embolus occludes in a cerebral vessel and obstructs blood flow to the brain. Approximately 800,000 people per year in the United States experience a new or recurrent stroke (1) and approximately 33% of these patients will die within 1 year post-stroke (39). Consequently, IS is a significant cause of morbidity, particularly in the elderly, where approximately half of these patients will have enduring moderate-to-severe neurologic deficits (40). Tables 1–3 provide a summary of the IL-10 preclinical and clinical IS studies described below.

**Table 1:** Summary of IL-10 ischemic stroke preclinical studies.

Experimental paradigm	Model	Outcomes	Animals	Reference
–	pMCAO	IL-10 is upregulated in neurons after stroke	Wistar	(41)
–	tMCAO	Hypertension blunts neuronal upregulation of IL-10	Wistar, spontaneously hypertensive rats	(41)
IL-10^−/−^	pMCAO	Larger infarct 24 h post-stroke in IL-10-deficient mice	C57BL/6	(42)
IL-10^−/−^	pMCAO	Larger infarct and increased neurologic deficits in IL-10-deficient mice	C57BL/10J	(43)
IL-10^−/−^, MOG_35-55_	tMCAO	CD4^+^ T cells reduce infarct through IL-10 secretion	C57BL/6	(44)
IL-10^−/−^, MOG_35-55_	tMCAO	Smaller infarct in WT mice treated with MOG_35-55_, benefit not seen in IL-10-deficient mice	C57BL/6	(45)
IV IL-10	pMCAO	IL-10 significantly reduces infarct volume	Spontaneously hypertensive rats	(46)
ICV IL-10	pMCAO	IL-10 significantly reduces infarct volume	Spontaneously hypertensive rats	(46)
ICV IL-10	pMCAO	IL-10 downregulates proinflammatory molecules and reduces infarct volume	C57BL/6J	(47)
Transgenic IL-10	pMCAO	Transgenics have smaller infarcts and reduced proinflammatory cytokines	C57BL/6J	(48)
Carotid AAV IL-10	tMCAO	Smaller infarct and less neuronal injury and neurological deficit scores with AAV treatment	Wistar	(49)
IM AAV IL-10	–	Reduced stroke incidence, prolonged survival with AAV treatment	Spontaneously hypertensive rats	(50)
H_2_S donor	tMCAO	H_2_S donors at reperfusion lead to increased IL-10 levels and BBB integrity	ICR	(51)
Transgenic IL-32α	tMCAO	IL-10 and STAT3 upregulation observed in mice with better outcomes	C57BL/6	(52)
Histone deacetylase inhibition	pMCAO	Treg activation is neuroprotective through IL-10 secretion	C57BL/6J	(53)
IP CD28SA	pMCAO	Treg amplification reduces infarct through increasing IL-10 levels	C57BL/6J	(54)
SQ G-CSF and SCF	pMCAO	Early and late treatment improves motor and cognitive function and promotes neurogenesis	C57BL/6, GFP-transgenic mice	(55)
SQ G-CSF and SCF	pMCAO	Early and late treatment increases IL-10 mRNA and reduces activated macrophages and microglia	C57BL/6	(56)
IV B-cells-expressing IL-10	tMCAO	Smaller infarct, less T-cell proliferation	C57BL/6J	(57)
μMT^−/−^, IV B-cells-expressing IL-10	tMCAO	IL-10-secreting B-cell replenishment in B-cell-deficient mice reduces infarct volumes	C57BL/6J	(58)
ICV Treg	tMCAO	Tregs act *via* IL-10 to promote neural stem cell proliferation	C57BL/6	(59)
ICV MSC	pMCAO	Increased IL-10 mRNA and protein levels, smaller infarct, decreased TNFα	Sprague-Dawley	(60)
ICV, IA, IV ADSC	tMCAO	Smaller infarct, improved neurological function, decreased TNFα	Sprague-Dawley	(61, 62)

*AAV, adeno-associated virus; ADSC, adipose-derived stem cells; CD28SA, CD28 superagonist antibody; G-CSF, granuloycyte colony-stimulating factor; IA, intra-arterial; ICV, intracerebroventricular; IM, intramuscular; IL-10, interleukin-10; IP, intraperitoneal; IV, intravenous; MSC, mesenchymal stem cells; SCF, stem cell factor; SQ, subcutaneous; Treg, regulatory T cell; μMT^−/−^, B-cell-deficient mice*.

**Table 2:** Summary of clinical ischemic stroke studies investigating IL-10 genetic polymorphisms.

Reference SNP ID	Population	Outcomes	Study modality	Reference
rs1800896	South Indian	Increased risk of IS	ARMS PCR	(63)
rs1800896	Chinese, Chinese, Chinese, Indian, Palermo	Increased risk of IS	Meta-analysis	(64)
rs1800896	Chinese	Increased risk of IS	ARMS PCR	(65)
rs1800896	Turkish	Increased risk of IS	RT-PCR	(66)
rs1800896	Chinese	Increased risk of IS, particularly in smokers	PCR-RFLP	(67)
rs1800896	Greek	Not associated with IS risk, but GG genotype predicts early stroke progression	RT-PCR	(68)
rs1800896	Meta-analysis	Not associated with IS risk, but associated with IS subtypes	Meta-analysis	(69)
rs1800872	Chinese	Increased risk of IS, lower serum levels of IL-10	ELISA, PCR-RFLP	(70)
rs3021094	Chinese	Increased risk of IS, lower serum levels of IL-10	ELISA, LDR	(70)
rs1554286	Chinese	Increased risk of IS, lower serum levels of IL-10	ELISA, LDR	(70)
rs1554286	Korean	Increased risk of hypertension, contributed to increased risk for IS	PCR	(71)
rs1518111	Korean	Increased risk of hypertension, contributed to increased risk for IS	PCR	(71)
rs1800871	Chinese, Chinese, Chinese, Palermo	Not associated with IS risk	Meta-analysis	(64)
627*C/*C	Russian	Associated with protection against hypertension	PCR	(72)
Promoter [ATA] Haplotype	Caucasian	Associated with lower serum IL-10 and risk of postoperative cardiovascular events in PAD	PCR	(73)

*ARMS, amplification refractory mutation system; ELISA, enzyme-linked immunosorbent assay; LDR, ligase detection reaction; PAD, peripheral artery disease; PCR, polymerase chain reaction; PCR-RFLP, polymerase chain reaction–restriction fragment length polymorphism; RT-PCR, real-time polymerase chain reaction; IL-10, interleukin-10; IS, ischemic stroke*.

**Table 3:** Summary of non-genetic IL-10 ischemic stroke clinical studies.

Study modality	Population	Outcomes	Reference
LPS whole blood assay	Netherland	Low IL-10 production in response to LPS increases risk for incident fatal IS	(74)
	Russian	Low serum IL-10 in IS and higher incidence of hemorrhagic transformation	(75)
ELISA	Canadian	Low plasma IL-10 and worse IS outcomes	(76)
ELISA	Brazilian	Low serum IL-10 and neurological deterioration	(77)
ELISA	Indian	IL-10 level is low within 72 h post-stroke, no correlation to NIHSS at admission	(78)
ELISA	Indian	Low IL-10 at 24 h but higher at 72 and 144 h for IS patients that survive compared with those that expire	(79)
ELISA	Chinese	IS patients have low IL-10 and Tregs at 7 and 28 days	(80)
ELISA	Eastern Finland	Plasma IL-10 level correlates with cardioembolic IS etiology	(81)
ELISA	Spaniards	High plasma IL-10 associated with SAI within 24 h	(82)
Immulite 1000	German	High IL-10 at 6 h predicts SAI	(83)
ELISPOT	Swedish	IS patients have elevated IL-10-secreting MNCs	(84)
–	Taiwanese	High serum IL-10 at 48 h and increased neurological impairment and adverse outcomes	(85)
–	Turkish	IL-10 not associated with IS prognosis	(86)

*ELISA, enzyme-linked immunosorbent assay; ELISPOT, enzyme-linked immunospot assay; LPS, lipopolysaccharide; MNC, mononuclear cells; NIHSS, National Institute of Health stroke scale; SAI, stroke-associated infection; Tregs, regulatory T cells; IL-10, interleukin-10; IS, ischemic stroke*.

### IL-10 in *In Vitro* Models

Cortical neuron cultures from IL-10^−/−^ mice are more susceptible to neurotoxicity following excitotoxicity and combined oxygen–glucose deprivation, and administration of exogenous IL-10 provides neuroprotection to cultures from both WT and knockout strains (42). This IL-10-mediated protection was later separately shown to be *via* the IL-10 receptor on cortical neurons and PI3K/AKT and STAT3 signal transduction pathways (87). The transcription factor Nrf2 is expressed widely throughout the body and upregulates the expression of numerous antioxidant genes in response to oxidative stress (88, 89). In astroglia preconditioned to oxidative stress, neuroprotection from oxidative stress–glucose deprivation was reported to be *via* an Nrf2/IL-10-dependent mechanism (90). Lipocalin-2 is significantly increased in neurons following stroke and is proposed to signal stress from injured neurons to supporting glia (91). Lipocalin-2 treatment of microglia results in glial activation, IL-10 release, and enhanced phagocytosis, and conditioned media from these microglia protect neurons from oxygen–glucose deprivation (91).

### IL-10 in Preclinical Models

In a permanent middle cerebral artery occlusion (MCAO) model, IL-10^−/−^ C57BL/6 mice have 30% larger infarct volumes compared with WT mice 24 h post-stroke (42). In this model, WT and IL-10^−/−^ mice display similar degrees of proinflammatory molecules within the first 6 h. However, after 4 days, the IL-10^−/−^ mice have higher levels of proinflammatory molecules that persist through the end of the first week after stroke (43).

Both systemic intravenous (IV) and central intracerebroventricular (ICV) exogenous administration of IL-10 reduces infarct volumes following permanent MCAO (46). Similarly, in a transgenic C57BL/6J mouse model overexpressing IL-10 (56% increase in the brain and 200% increase in the plasma), infarct volumes were reduced by 40% 4 days following permanent MCAO (48). In the same study, these findings were associated with upregulation of free radical scavengers such as glutathione and manganese superoxide dismutase, reduced activity of the proapoptotic protein caspase 3, and downregulation of IL-1β, IFN-γ, and TNFα 1 day post-stroke (48). Injection of a recombinant adeno-associated viral vector serotype 1 expressing IL-10 into the cerebral artery of rats 3 weeks prior to MCAO results in elevated IL-10 serum levels 3 weeks after injection and decreases neurologic deficit scores, infarct volume, and neuronal injury (49). Systemic IL-10 overexpression in stroke-prone spontaneously hypertensive rats reduces the incidence of stroke, decreases stroke-associated symptoms, and improves survival (50). In Wistar rats, IL-10 is upregulated in viable neurons in the ischemic brain following permanent and transient MCAO, and hypertension blunts this response, potentially contributing to the worse outcomes in the hypertensive setting (41).

Interleukin-10 appears to exert its anti-inflammatory effects in part by downregulation of NFκB (92). Administration of hydrogen sulfide donors at the time of reperfusion protects BBB integrity after ischemia/reperfusion and is accompanied by enhanced IL-10 expression, reduced NFκB nuclear translocation, and MMP-9 and NOX4 activity (51). Moreover, upregulation of IL-10 and the STAT3 pathway and downregulation of NFκB have been proposed as the mechanisms for improved outcomes following cerebral ischemia in mice that overexpress IL-32α (52). A recent whole genome array in C57BL/6 mice showed that stroke upregulated 347 genes 24 h after reperfusion. In this study, ICV injection of IL-10 5 min after occlusion downregulated 341 of these genes (47).

Immune cells, including T and B cells, are important in attenuating neuroinflammation *via* the modulation of various cytokines and chemokines, with IL-10 playing a central immunomodulatory role (53, 58). Tolerizing mice to oligodendrocyte glycoprotein prior to MCAO reduces infarct size by 70% at 24 h and by 50% at 72 h, but this effect was not seen in IL-10^−/−^ mice (45). This protection is specifically mediated by IL-10 secreted from CD4^+^ T cells, which were able to protect mice from stroke only when originating from WT mice (44). Elevated levels of IL-10 also reduce the number of CD11b^+^ cells that may contribute to secondary infarct expansion *via* nitric oxide pathways (45). Histone deacetylase plays a role in activating the expression of Foxp3 on Tregs, which secrete IL-10, and IL-10 was suggested as the main mediator of attenuated infarct volume and behavioral deficits, reduced levels of proinflammatory cytokines, and increased number of Tregs in the brain of MCAO mice with histone deacetylase inhibition (53). Similarly, expansion of the Treg cell population in the CNS *via* administration of a CD28 superagonist monoclonal antibody at onset of reperfusion reduced infarct size 7 days following MCAO, and its effect was attributed to an increased amount of IL-10 (54). Transferring IL-10-producing B cells into B cell-deficient mice 24 h after MCAO reduced infarct size, the amount of T cells and monocytes in the brain parenchyma, and the peripheral proinflammatory milieu (58). A follow-up to this study showed similar results in B cell-sufficient mice (57). Notably, IL-10-producing B cells also upregulated the number of Tregs (58), perhaps indicating a positive feedback loop between B cells and Tregs, both of which are neuroprotective *via* IL-10 production.

Neurogenesis is a highly debated topic for potentially enhancing neural recovery following IS. Injection of activated Tregs into the lateral ventricle of C57BL/6 mice after 60 min of transient MCAO promotes neural stem cell proliferation in the subventricular zone in ischemic and normal mouse brains. However, this effect was eliminated by blocking IL-10 with a neutralizing antibody, indicating that activated Tregs act *via* IL-10 to promote neural stem cell proliferation (59). Hematopoietic cytokines such as GCSF and stem cell factor have been shown to promote neurogenesis (55) and also may be responsible for providing the initial signals to produce IL-10 in the setting of IS (56). Treatment with these cytokines early (1–10 days) and later (11–20 days) after MCAO markedly elevates IL-10 mRNA levels, reduces the levels of activated microglia/macrophages, and does not change proinflammatory cytokine expression in C57BL/6J mice (56). Another study where bone marrow-derived mesenchymal stem cells were transplanted into the lateral ventricle of Sprague-Dawley rats before permanent MCAO afforded similar results, where IL-10 mRNA and protein levels are increased for up to 4 days post-stroke, TNFα is decreased, infarct volumes are smaller, and neurologic function is preserved (60). Adipose tissue-derived stem cells trigger similar effects as mesenchymal stem cells (61) and are most effective when delivered intravenously at 24 h after MCAO (62). Thus, either administration of stem cells themselves or hematopoietic cytokines seem to improve outcomes following IS in part through increasing IL-10 levels.

### IL-10 in Clinical Studies

Several epidemiologic studies have analyzed the genetic contribution of IL-10 to IS risk and outcome. A study in a Chinese population showed that lower basal serum levels of IL-10, an IL-10 promoter single nucleotide polymorphism (SNP) rs1800872 (AA vs. AC + CC), and IL-10 intron SNPs rs1554286 (TT vs. CT + CC) and rs3021094 (CC/CA vs. AA) are all associated with higher risk for IS (70). Similarly, subjects with low IL-10 production in response to LPS stimulation have a higher risk for fatal stroke (74). A study in a South Indian population showed an increased risk of IS with the A allele of the IL-10 promotor SNP rs1800896 (A vs. G), which is associated with low IL-10 production (63). In agreement, a recent meta-analysis showed that the IL-10 1082G/A SNP (rs1800896) is associated with increased risk of IS, but not the 819 C/T SNP (rs1800871) (64, 65). Several other studies further revealed that the IL-10 1082G/A SNP is associated with susceptibility for an IS (66, 67, 93, 94). However, in a different study, the IL-10 1082G/A SNP was not associated with the occurrence of an IS, but the GG genotype predicted early stroke progression and functional dependency independent of other standard risk factors (68). Similarly, another study showed that the IL-10 1082G/A SNP was significantly associated with the risk for specific subtypes of IS, but no significant association was found with the overall risk for an IS (69). A study in a Korean population showed that rs1518111 and rs1554286 are not associated with IS *per se*, but are associated with hypertension in the risk of IS (71). Similar results were seen in a Russian population, where the IL-10 627*C/*C genotype is protective against hypertension in male patients with stroke (72). One other study in an Eastern Finland population showed that IL-10 plasma concentration independently correlates with cardioembolic high-risk sources, suggesting its usefulness in improving diagnosis of stroke etiology (81). Lastly, in Caucasian patients with peripheral artery disease receiving elective revascularization, the IL-10ATA haplotypes that are associated with lower serum IL-10 levels correlate with a high risk for postoperative cardiovascular events (73).

The relative levels of IL-10 at various times following IS differ from controls and appear to have important implications on IS patient outcomes. For example, at 48 h after IS, mean serum IL-10 is significantly higher than in healthy or at-risk controls, and high IL-10 sera levels independently correlate with severe neurologic deficits (NIHSS ≥12) at 48 h post-stroke and predict major adverse clinical outcomes (recurrent IS, any cause of death, NIHSS ≥12) at 90 days (85). However, in another study, IL-10 levels within the first 72 h after IS were lower than in controls and no significant correlation with NIHSS at admission was observed (78). As compared to those who die, IS patients who improve have been shown to have lower IL-10 at 24 h, but increased levels at 72 h and 144 h (79). In other studies, low IL-10 levels are associated with acute neurologic decline post-stroke (76, 77). Yet another small study found no correlation with IL-10 and other cytokines and IS patient prognosis (86). IS patients with low levels of IL-10 had a higher incidence of hemorrhagic transformation (75), perhaps providing a link between IL-10 and endothelial integrity. At 7 and 28 days, IS patients appear to have lower number of Tregs and IL-1 and higher relative levels of proinflammatory cytokines such as IL-6 and IL-1β (80). In the Leiden 85-plus study, patients with a history of stroke displayed lower IL-10 production in response to LPS stimulation than subjects without stroke (74). No correlation between IL-10 levels and infarct volume has been found (81, 86). Considerable variation exists among the aforementioned studies and is likely due to the different study populations, relatively small cohort sizes, differences in study methodology, and the consistency or lack thereof for identifying and correcting for covariates. Additional large and controlled prospective studies are needed to establish the link between IL-10 and IS severity, recovery, and outcomes.

Ischemic stroke may induce a functional immunosuppressive state that may persist for several weeks, wherein patients may be more susceptible to infection, and infection is a leading cause of the high mortality rates seen long after an IS (82, 95). The temporal IL-10 profile appears to be different between those that develop stroke-associated infection (82, 83). Two studies have shown that high IL-10 levels at admission or 6 h following ischemia is an independent predictor of infection (82, 83). In one of those studies, TNFα and the TNFα/IL-10 ratio was decreased (82). Another study found that the levels of IL-10-secreting mononuclear cells (MNCs) were highly elevated in IS patients compared with healthy individuals (84). Thus, the immunomodulatory role of IL-10 may be an important consideration in understanding infectious processes and mortality after IS.

### Summary and Comparison of the Role of IL-10 in Preclinical and Clinical IS Studies

Ischemic stroke invokes a robust local inflammatory response in the brain that significantly contributes to secondary brain damage and poor outcomes. With IL-10 representing a canonical anti-inflammatory cytokine that intriguingly has a special role in the resolution of inflammation, it is not surprising that many studies investigating the role of IL-10 in IS have been performed. *In vitro* models have provided evidence for a neuroprotective function of IL-10 under hypoxic and excitotoxic conditions. In IS preclinical models, IL-10 reduces infarct volume *via* its protective effects on the vascular endothelium and attenuation of inflammatory cascades. Multiple epidemiologic genetic studies have in general shown that polymorphisms in the IL-10 gene that lower IL-10 levels increase the risk of IS. However, clinical studies measuring IL-10 levels after IS are more varied with regard to the role of IL-10 in predicting neurologic outcomes and complications. Even with all of the studies described here, it is clear that additional work is needed to clarify the role of IL-10 in predicting the risk for IS, modulating IS outcomes, and whether IL-10 has biomarker and/or therapeutic potential after IS.

## Subarachnoid Hemorrhage

Subarachnoid hemorrhage affects an estimated 7.2–9.0 per 100,000 people/year in the United States (96), and most cases are due to the rupture of a cerebral aneurysm resulting in bleeding within the subarachnoid space (97). Although approximately 15% of people die within a few hours from the bleed and a proportion will never make it to a hospital (98), even those who initially survive the bleed can have significant morbidity and mortality. In addition to early brain injury that occurs at the time of SAH, patients can also develop neurological deficits or die from delayed cerebral ischemia (DCI), which is often secondary to cerebral vasospasm (CV) (99, 100). Among the possible mechanisms for CV and DCI (13, 100–110), inflammation is a common element. IL-10 has not been extensively studied in the context of SAH, despite its proposed pleiotropic immunomodulatory effects relevant to SAH pathophysiology. Tables 4 and 5 provide a summary of the IL-10 preclinical and clinical SAH studies described below.

**Table 4:** Summary of IL-10 subarachnoid hemorrhage preclinical studies.

Experimental paradigm	Model	Outcomes	Animals	Reference
–	Cisternal Autologous Blood	Non-significant change in IL-10 mRNA levels in the basilar artery	Mongrel Canines	(111)
ICV ACh	Cisternal Autologous Blood	Non-significant change in CSF IL-10 levels; ACh had no effect on IL-10 level	Sprague-Dawley	(112)
–	Endovascular Perforation	Non-significant change in IL-10 mRNA levels in the cortex	Wistar	(113)

*ACh, acetylcholine; ICV, intracerebroventricular; IL-10, interleukin-10*.

**Table 5:** Summary of IL-10 subarachnoid hemorrhage clinical studies.

Study modality	Population	Outcomes	Reference
–	Austrian	Mean blood IL-10 levels low throughout ICU stay	(114)
ELISA	Swedish	Microdialysate IL-10 present at low levels and remained constant through 7 days post-bleed	(115)
ELISA	British	Plasma and CSF IL-10 levels constant through 10 days post-bleed, plasma and CSF levels equal	(116)
PCR	American	IL-10 mRNA not found in aneurysm walls, but was present in temporal artery controls	(117)
TaqMan Allelic Assay	Indian	IL-10 SNPs rs1800871 and 1800872 associated with intracranial aneurysm incidence, no correlation with rupture	(118)

*ICU, intensive care unit; PCR, polymerase chain reaction; SNP, single nucleotide polymorphism; IL-10, interleukin-10*.

### IL-10 in Preclinical Models

Temporal changes in IL-10 are seen following experimental SAH, although these are not statistically significant (111–113). After autologous blood infusion into the cisterna magna of canines, IL-10 mRNA expression in the basilar artery tends to be increased for up to 14 days post-ictus (111). Similarly, IL-10 tends to be increased in the CSF at 6 h and then declines, but remains elevated through 48 h after autologous blood injection into the cisterna magna of male adult Sprague-Dawley rats (112). Interestingly, although acetylcholine was able to attenuate CV in this model, it had no effect on IL-10 levels (112). Last in an endovascular perforation model of SAH in Wistar rats, trends toward higher IL-10 mRNA in the cortex are seen at 48 h (113). In contrast to the less impressive changes in IL-10, these studies have shown highly increased levels of proinflammatory cytokines such as IL-1α (111), IL-1β (113), IL-6 (111, 112), IL-8 (111), and TNFα (111) at 48 h following experimental SAH. Indeed, Aihara and colleagues postulated that the anti-inflammatory effects of IL-10 were overwhelmed by these proinflammatory cytokines such that IL-10 was unable to counteract the deleterious inflammatory processes involved in CV pathophysiology and thereby improve SAH outcomes (111).

### IL-10 in Clinical Studies

Studies in humans have also not shown significant changes in IL-10 after SAH (114–117). One study reported that IL-10 mRNA expression was virtually non-existent in aneurysm walls, although it was present in the walls of temporal artery controls (117). In microdialysate, when detectable, IL-10 was present in extremely small concentrations and remained at relatively constant levels through 7 days post-bleed (115). Similarly, IL-10 levels were constant through at least 10 days post-bleed in paired plasma and CSF samples, and the levels were not significantly different between the CSF and plasma (means were below 10 pg/ml) (116). In another study, mean IL-10 blood levels throughout the intensive care unit stay were also quite low (mean ≈11 pg/ml) (114). However, a study in an Indian population showed that IL-10 SNPs rs1800871 and rs1800872 are significantly associated with the incidence of intracranial aneurysms, independent of epidemiological factors, although it is unclear whether this increases the risk for aneurysm rupture and thus the incidence of SAH (118). Additional larger controlled studies are necessary to clarify the association of IL-10 with aneurysm formation and rupture and the role of IL-10 after SAH, including its prognostic and diagnostic predictive potential for SAH outcomes.

### Summary and Comparison of the Role of IL-10 in Preclinical and Clinical SAH Studies

In comparison with the other forms of acute brain injury described herein, substantially fewer studies have explored the contribution of IL-10 in the setting of SAH. Preclinical studies have shown non-significant increases in IL-10 after SAH, accompanied by robust changes in proinflammatory cytokines, suggesting an imbalance in the inflammatory milieu that could possibly contribute to prolonged disease course and worse SAH outcomes. In addition, clinical studies have not shown significant changes in IL-10 after SAH, but no studies have investigated whether IL-10 affects clinical outcomes or complications such as CV and/or DCI, or whether IL-10 has therapeutic or biomarker potential. It is clear that additional work is needed to understand how IL-10 changes in the various biocompartments (serum, CSF, brain) after SAH and the resulting implications on SAH pathophysiology.

## Intracerebral Hemorrhage

Intracerebral hemorrhage is characterized by bleeding within the brain parenchyma and can result from various etiologies such as trauma, vascular malformations, medical therapies such as anticoagulants, amyloid angiopathy, and most commonly, hypertension. ICH has a poor prognosis, and there are currently no therapies to decrease the 30-day mortality rate of 35–50% (119). At present, the only available interventions include supportive care, and in some select cases, invasive surgery to evacuate hematomas (120), yet, it is well established that inflammation is a key player in the overall brain damage and edema after ICH that results in enduring neurological deficits. Tables 6 and 7 provide a summary of the IL-10 preclinical and clinical ICH studies described below.

**Table 6:** Summary of IL-10 intracerebral hemorrhage preclinical studies.

Experimental paradigm	Model	Outcomes	Animals	Reference
–	Collagenase	IL-10 increased at 6 h and 7 days	Sprague-Dawley	(121)
Atorvastatin	Collagenase	Atorvastatin treatment leads to both dose-dependent increases in IL-10 and decreases in TNFα	Sprague-Dawley	(122)
CD36^−/−^	Autologous	CD36 deficiency leads to decreased perihematoma IL-10 levels	C57BL/6	(123)

*LPS, lipopolysaccharide; TGFβ, transforming growth factor-β; TNFα, tumor necrosis factor alpha; IL-10, interleukin-10*.

**Table 7:** Summary of IL-10 intracerebral hemorrhage clinical studies.

Study modality	Population	Outcomes	Reference
ELISA	Japanese	Higher plasma IL-10 levels in ICH patients with poor 1 month old mRS outcomes	(124)
ELISA	Taiwanese	Higher plasma IL-10 level on admission associated with hematoma expansion and worse 1 month old outcome	(119)
ELISA	Polish	Admission IL-10 levels negatively correlate with GCS, IL-10 correlates with IL-6 levels	(125)
ELISA	Polish	Systemic IL-10 at 2 days is higher in left hemisphere hemorrhages compared to right hemisphere hemorrhages	(126)
ELISPOT	Swedish	IL-10-secreting MNCs are elevated compared to healthy controls	(84)

*ELISA, enzyme-linked immunosorbent assay; ELISPOT, enzyme-linked immunospot assay; GCS, Glasgow Coma Scale; HPLC, high-performance liquid chromatography; MNCs, mononuclear cells; mRS, modified Rankin scale; IL-10, interleukin-10*.

### IL-10 in Preclinical Models

Microglia, a primary player in immune surveillance and the initiation of inflammation in the CNS (127), become activated shortly after ICH (12) and secrete proinflammatory cytokines such as TNFα, IL-6, and IL-1β (127). These microglia also secrete IL-10, which acts on astrocytes in the brain to redirect their focus from the production of a proinflammatory cytokine profile toward the production of TGFβ (127). TGFβ then feeds back to act on the microglia and attenuate their proinflammatory response. IL-10 can also increase CD36 expression and thereby enhance the erythrophagocytic ability of microglia, and with CD36 deficiency, mRNA levels of TNFα and IL-1β are higher and IL-10 levels are significantly lower in the perihematomal tissues (123). Additionally, IL-10 levels are significantly increased at 6 h and 7 days after ICH with respect to sham, suggesting that IL-10 may have early and late influences on ICH outcomes (121).

Interestingly, a study in IL-10^−/−^ mice suggests that the presence of IL-10 is protective against the development of cerebral hemorrhage (128). Cerebral hemorrhage and edema are attenuated by anti-TNFα therapy (128), indicating that the main protective effect of IL-10 is derived from its inhibition on the production of proinflammatory cytokines, namely, TNFα (129–131). This same effect is found in adult male Sprague-Dawley rats, where statins (2, 5, 10 mg/kg) produce dose-dependent increases in IL-10 levels and are associated with dose-dependent decreases in TNFα, as well as fewer activated microglia, sensorimotor deficits, and inflammation and less edema 3 days after ICH (122).

### IL-10 in Clinical Studies

Only a couple of studies have looked at IL-10 genetic polymorphisms and the risk for developing ICH. The IL-10 1082G/A SNP has been identified as a risk factor for ICH in a North Indian population (132). This SNP was not associated with the risk for hemorrhagic presentation of brain arteriovenous malformations (133).

Interleukin-10 and the amount of IL-10-secreting MNCs are elevated in peripheral blood 2 days following ICH (84, 125). Another study found that systemic IL-10 release was significantly higher in ICH patients with left hemisphere hemorrhage compared with those with right hemisphere hemorrhage at 2 days, whereas no such correlation was found for IL-6 (126). Because dysfunction of the autonomic nervous system is a common ICH complication (134), the autonomic nervous system can be regulated asymmetrically, and the sympathetic nervous system can induce a notable increase in systemic IL-10 release, this study posits autonomic dysfunction as a potential mechanism for asymmetrical regulation of IL-10 after ICH (126).

At admission, IL-10 levels are negatively correlated with the Glasgow Coma Scale (GCS), although a stronger negative correlation was found for IL-6 and GCS (125). At 2 days post-bleed, IL-10 levels correlate with IL-6 levels, and IL-6 levels correlate with hematoma volume and mass effect, but IL-10 does not (125). Hematoma expansion is a major cause of morbidity and mortality after ICH, and inflammation may be associated with its pathogenesis (119). In spontaneous ICH, higher plasma IL-10 levels on admission are associated with hematoma expansion and worse 30-day outcomes (119). Another study also found that plasma IL-10 levels were higher in ICH patients with poor outcome (modified Rankin Scores of 3–6) at 1 month (124), although this study did not utilize a multivariate model.

### Summary and Comparison of the Role of IL-10 in Preclinical and Clinical ICH Studies

Similar to IL-10 and SAH, few studies have explored the contribution of IL-10 in the setting of ICH, although IL-10 appears to be elevated following experimental and clinical ICH. In preclinical models, IL-10 exerts a protective effect against spontaneous cerebral hemorrhage by downregulating TNFα, a key proinflammatory cytokine, and some weak evidence exists suggesting that IL-10 is neuroprotective after ICH. Clinically, very little is evident regarding the contribution of IL-10 polymorphisms. However, other studies generally have found that IL-10 levels correlate with the extent of initial brain injury and have prognostic value, suggesting both diagnostic and prognostic biomarker potential for IL-10 after ICH.

## Traumatic Brain Injury

Traumatic brain injury can result from a direct force like a concussion, an indirect force from a blast injury, or a penetrating injury. TBI is a prevalent clinical problem (9), with an estimated two million people in the United States sustaining a TBI each year (135, 136), leading to an astounding estimated 30.5% of injury-related deaths (137). TBI has substantial long-term effects on patients (137, 138) and imposes a significant financial burden (9, 135, 136, 138). The outcome for a TBI patient is determined by the severity of the initial injury, which is related to the mechanism of primary injury and degree of physical force, and secondary injury, linked to neuroinflammation (8, 9, 139). It is proposed that the majority of damage from TBI is the product of secondary damage, which is closely associated with the production and recruitment of proinflammatory cytokines and other inflammatory mediators (8, 140). Tables 8 and 9 provide a summary of the IL-10 preclinical and clinical TBI studies described below.

**Table 8:** Summary of IL-10 traumatic brain injury preclinical studies.

Experimental paradigm	Model	Outcomes	Animals	Reference
–	Weight Drop	IL-10 mRNA increases immediately post-injury, IL-10 protein elevated at 2 h	Sprague-Dawley	(135)
IL-10 Gelfoam	Corticectomy	Local IL-10 administration reduces reactive astrocytes and TNFα at 4 days	CD1	(141)
IL10^−/−^ and SQ IL-10	CCI	IL-10 treatment at 1 h reduces lesion volume, edema, and improves motor and cognitive function at 5d	C57BL/6	(142)
IP IL-10	CCI	IL-10 treatment reduces inflammation at 5 h, but does not affect cognitive or motor function	Sprague-Dawley	(143)
IP Triptolide	CCI	Triptolide treatment increases brain IL-10 levels at 1d and improves anatomical and neurobehavioral outcomes	Sprague-Dawley	(144)
–	Repetitive mTBI	IL-10 lower at days 1, 3, 7, 14, and 30 compared to single mTBI	Sprague-Dawley	(145)
IL10^−/−^ and HBOT	CCI	HBOT increases serum and ipsilateral cortex IL-10 levels, reduced lesion volume, and improved outcome in WT mice	C57BL/6	(142)
HBOT	Fluid Percussion	HBOT increases IL-10 level at 4 days and stimulates angiogenesis and neurogenesis	Sprague-Dawley	(146)
IV, SQ, SCV IL-10	Fluid Percussion	IL-10 treatment (SQ and IV) improves neurological recovery	Sprague-Dawley	(147)

*CCI, controlled cortical impact; HBOT, hyperbaric oxygen therapy; ICV, intracerebroventricular; IP, intraperitoneal; IV, intravenous; mTBI, mild traumatic brain injury; SQ, subcutaneous; TNFα, tumor necrosis factor alpha; IL-10, interleukin-10*.

**Table 9:** Summary of IL-10 traumatic brain injury clinical studies.

Study modality	Population	Outcomes	Reference
Multi-analyte assay	British	Plasma IL-10 levels peak between days 5 and 6	(148)
Multiplex bead array assay	American	IL-10 elevated compared to controls, no correlation with initial GCS, age, gender, or outcome	(130)
Multiplex bead array assay	Brazilian	Elevated serum IL-10 levels correlates with GCS and hospital mortality in severe TBI patients	(149)
Multiplex bead array assay	American	Plasma IL-10 levels elevated at 6 months old and correlate with GOS at 6 and 12 months old	(137)
Multiplex bead array assay	American	Serum IL-10 levels elevated in trauma patient with and without TBI	(150)
ELISA	German	Plasma IL-10 levels peak within 3 h	(151)
ELISA	German	Plasma IL-10 levels elevated within 3 h	(152)
ELISA	Swiss	IL-10 levels elevated up to 22 days post-injury and second peak in concentration seen	(131)
ELISA	Japanese	CSF IL-10 elevated for 24 h post-injury before declining	(153)
ELISA	American	IL-10 elevated in pediatric TBI relative to controls at days 1–3 and predicts mortality, no correlation with GCS	(154)
ELISA	Swedish	Temporal IL-10 pattern has no initial peak and no gradual decrease from 2 to 7 days	(115)
ELISA	Japanese	CSF IL-10 is better predictor of outcomes after TBI with extracranial injuries compared to serum IL-10 levels	(155)
ELISA	Polish	Isolated head injury vs. those with accompanying injury results in no difference in serum IL-10 level	(129)
ELISA	German	No correlation between BBB disruption in TBI and serum or CSF IL-10 level	(139)
Immulite	German	High initial CSF IL-10 that decreases over time and serum IL-10 that increases over time predicts mortality	(156)

*GCS, Glasgow Coma Scale; GOS, Glasgow Outcome Scale; ELISA, enzyme-linked immunosorbent assay; IL-10, interleukin-10; TBI, traumatic brain injury; BBB, blood–brain barrier*.

### IL-10 in Preclinical Models

Understanding the temporal relationship between the injury and elaboration of IL-10 provides insight into its immunomodulatory role after TBI. A study in adult male Sprague-Dawley rats found that brain IL-10 mRNA expression increases immediately following TBI, whereas IL-10 protein levels are stable initially, and only begin to rise rapidly after 2 h (135). These findings suggest that the surge in IL-10 levels is due to an increase in local IL-10 synthesis rather than systemic IL-10 entering through a leaky BBB. IL-10 protein levels continue to rise through 24 h, although at a slower rate (135). No change in plasma IL-10 levels is seen throughout this timeframe (135). Following repetitive mild TBI, TNFα and IL-6 are higher, and the expression of IL-10 is lower when compared to a single TBI group on days 1, 3, 7, 14, and 30 post-injury (145).

Studies with IL-10^−/−^ mice have suggested that IL-10 is beneficial after TBI, although IL-10 administered after TBI as a therapeutic agent has provided mixed results depending on the experimental paradigm and administration route. At 4 weeks after TBI, 8-week-old IL-10^−/−^ C57BL/6 female mice have larger lesion volumes, increased edema and inflammation, worse motor and cognitive function, and higher levels of BBB dysfunction and apoptosis after controlled cortical impact-induced TBI (142). Additionally, using the lateral fluid percussion TBI model, SQ IL-10 (100 µg) administered 1 h after TBI results in a significant reduction in lesion volume and edema at 5 days, as well as improved recovery of motor and cognitive function (142). In another study using the lateral fluid percussion model and adult male Sprague-Dawley rats, both SQ and IV IL-10 (100 µg) improved neurological recovery, but ICV dosing (1 or 6 µg) did not (147). Although not statistically significant, survival rates in the SQ and IV groups were higher than in the controls, and the ICV group had dose-dependent lower survival rates (147). IV administration also reduced TNFα and IL-1β expression in the injured cortex and hippocampus (147). Although these studies have shown that systemic IL-10 treatment reduces neuroinflammation and improves neurological recovery (142, 147), a different study found that while systemically administered IL-10 (5 µg) does reduce inflammation (75% reduction in neutrophil accumulation) at 5 h, there is no improvement in motor or cognitive recovery (143). Last, central IL-10 administration following corticectomy in adult female CD1 mice results in a reduction in the number of reactive astrocytes and TNFα levels at 4 days (141), corroborating previous studies showing a reduction in neuroinflammation with IL-10 treatment.

A couple of studies with drugs or therapeutic regimens have demonstrated improved TBI outcomes through increased IL-10 protein levels and IL-10-dependent mechanisms. TBI has a significant ischemic injury component associated with poor cerebral blood flow (157). Hyperbaric oxygen (HBO) therapy has been suggested to provide some therapeutic benefit in conditions where poor blood flow and hypoxia lead to secondary tissue injury, and HBO therapy following TBI improves outcomes (142, 158). HBO therapy increases IL-10 at 4 days in adult Sprague-Dawley rats and mice above the levels induced by TBI alone in both the lateral fluid percussion (146) and the controlled cortical impact (142) models. The reported beneficial effects of HBO therapy after TBI are thought to be through an IL-10-dependent mechanism, as the smaller lesion volumes, less edema, improved motor and cognitive recovery, decreased inflammation, reduced apoptosis and BBB dysfunction 4 weeks after TBI with HBO therapy are only seen in WT mice and not in IL-10^−/−^ mice. Triptolide, an anti-inflammatory molecule, given immediately after TBI significantly increases IL-10 levels in the brain after 1 day and attenuates increases in proinflammatory cytokines; improves neurobehavioral outcomes; and reduces edema, contusion volume, and apoptosis (144).

### IL-10 in Clinical Studies

The temporal profile of IL-10 in clinical TBI studies is not conclusive. Early reports found that plasma IL-10 levels peak within the first 3 h (151, 152), while a later study showed the peak is between 5 and 6 days post-injury (148). IL-10 levels may remain elevated for up to 22 days (131, 139) or even up to 6 months (137), and in some cases, there is a second peak in concentration (131, 152). In contrast to this general pattern of a rise in IL-10 levels followed by a gradual decline in the CSF and plasma (153), others have been unable to identify any pattern of IL-10 levels in microdialysate (115). Another study in patients with a severely disrupted BBB found high initial CSF IL-10 levels that decreased over time and serum IL-10 levels that increased over time (156), supporting a heightened intrathecal IL-10 synthesis after TBI with overflow of the cytokine into the systemic circulation (137), augmenting baseline systemic IL-10 levels. However, there is inconsistency in the literature as to whether IL-10 levels are more dramatically increased in the CSF or serum following TBI (131, 139), rendering it difficult to determine the source of increased IL-10 levels. This discrepancy likely relates to isolated head injury versus multiple injuries, as one study found that serum levels were higher than CSF levels in patients with additional injuries; however, in patients with isolated TBI, CSF concentrations were greater than or equal to the corresponding serum levels (155). Isolated head injuries have also been shown to result in either no difference in serum IL-10 levels (129) or lower IL-10 levels than that seen with multiple injuries (151, 152). Moreover, serum levels of IL-10 are elevated in trauma patients with and without brain injury (150, 159). Thus, the heterogeneous nature of clinical TBI patients may be in part responsible for the inconclusive patterns and biocompartmental distribution of IL-10 following TBI.

Similarly, it seems that clinical severity measures and TBI complications such as GCS, various Injury Scores/Scales, and BBB dysfunction/disruption have not been definitively correlated with IL-10 levels. Although most studies found no correlation with GCS (129, 130, 153–155) or Injury Severity Score (129, 155) and IL-10 levels, a minority endorse a significant association (149, 151). Blood–brain barrier BBB dysfunction/disruption is one component of secondary brain damage after TBI (8, 135, 139, 147, 160), and although more severe BBB dysfunction is evident in non-survivors of TBI (156), no significant correlation can be made between IL-10 levels in either the serum or CSF and the degree of BBB disruption (131, 139, 156). Disruption to BBB integrity often contributes to elevated intracranial pressure (ICP), another common complication of TBI (140, 161) that is associated with poor outcomes (136, 140). Although high ICP coupled with sympathetic activation has been shown to increase systemic IL-10 levels (162), and some studies have demonstrated higher IL-10 levels in patients with both high ICP and unfavorable outcomes (155, 156), not all studies have found such an association between ICP and IL-10 levels (131, 150). Although some interleukins exhibit a stronger and more prolonged response in females (115), no studies have found a clear association between IL-10 levels and gender (115, 130). One study has shown that increased IL-10 levels after TBI are associated with age, such that increased levels after TBI are found with ages less than 4 years (154).

Interleukin-10 is elevated after TBI and exhibits a more prolonged response when compared to other cytokines (115), and although many have attempted to correlate IL-10 levels with outcomes, this effort has not been reliably successful (129, 130, 136, 139, 150, 153, 163–166). However, a few studies did find that elevated IL-10 concentrations are associated with unfavorable outcomes (137, 149, 154–156). Higher CSF IL-10 in pediatric patients (154) and elevated serum and CSF IL-10 levels in adult patients were significantly associated with mortality (156). Non-survivors demonstrate higher serum IL-10 levels at admission that continued to rise over the next 24 h, whereas survivors have lower IL-10 serum levels at admission that subsequently decrease (156). Additionally, non-survivors have higher CSF IL-10 levels at admission; however, both the non-survivors and survivors demonstrate decreasing CSF IL-10 levels over time (156). In another study, elevated IL-10 levels at 10 or 30 h after TBI were 6 and 5 times more frequently associated with hospital mortality, independent of GCS, age, and systemic trauma (149). It appears that early IL-10 correlates best with outcome, as both admission CSF and serum IL-10 levels within the first 30 h correlate with mortality, whereas later time points do not (149, 156). Increased CSF and serum levels correlate with a GCS score less than 4 (137, 155). Additionally, in a multivariate analysis, one study found that a higher IL-6/IL-10 ratio from 2 weeks to 3 months was significantly associated with a GCS score less than 4 at 6 months (78). Hypothermia is known to attenuate the proinflammatory response following TBI^147^; however, monitoring of IL-10 levels in severe TBI patients divided into hypothermia and normothermia treatment groups revealed no significant differences in CSF or serum IL-10 levels, nor outcomes in pediatric or adult patients (130, 153, 167).

It is well documented that IL-10 levels in humans and experimental models increase in both the serum and CSF shortly after TBI (8, 129, 135, 151, 152, 168) and remain elevated for many days (131, 152) followed by a slow decline. However, due to methodological variations in studies, the prognostic and diagnostic value of this cytokine remains unclear.

### Summary and Comparison of the Role of IL-10 in Preclinical and Clinical TBI Studies

Traumatic brain injury is a heterogenous type of acute brain injury that involves a complex interplay of both direct primary injury and secondary injury, the latter of which is closely linked to neuroinflammatory processes. Preclinical studies have shown that IL-10 reduces neuroinflammation following brain trauma and, in general, IL-10 treatment improves neurological outcomes after TBI. Clinically, several studies have shown that IL-10 levels increase after TBI, although the temporal profile of IL-10 levels and whether IL-10 correlates with initial injury severity is less clear. Regarding the prognostic potential, it appears that IL-10 has the most utility in predicting mortality after TBI, although given the controversy in the literature, future work is necessary to further define the role of IL-10 in predicting other outcomes.

## Conclusion

Interleukin-10 is significantly elevated following brain injury and appears to play a variety of roles depending on the type of acute neurologic insult, where it interacts with each condition’s overlapping, yet distinct, pathophysiology and secondary complications. High IL-10 levels tend to predict worse outcomes after hemorrhagic brain injury, whereas the converse is true for brain ischemia, low IL-10 levels resulting from SNPs increase the risk for IS and low levels after IS predict worse outcome. While IL-10 appears to have prognostic value, comparatively far fewer studies report on diagnostic potential. In the limited work in the literature, significant controversy exists where some report no correlation with measures of initial brain injury severity and others report significant correlations. From a therapeutic perspective, preclinical models have shown that IL-10 administration after IS and TBI lend better outcomes, although no work has been done in this area for SAH or ICH. Intertwined with all these acute pathologic processes are the effects of IL-10 on the vasculature, where it is crucial for protection. Finally, the varied effects and roles of IL-10 after IS, SAH, ICH, and TBI likely stems from pathology-dependent differences in the temporal balance of pro- and anti-inflammatory mediators. The latter point is particularly important in the context of IL-10, given its pleiotropic immunomodulatory functions that polarize the inflammatory system to an anti-inflammatory phenotype, aiding in the resolution of the neuroinflammation. It is likely that the ability of IL-10 to overcome the proinflammatory milieu is temporally different between the various forms of acute brain injury, with delays resulting in prolonged inflammation that exacerbates secondary brain damage, leading to worse outcomes. Novel therapies targeted to control inflammation will hinge on an understanding of the complex balance of the pro- and anti-inflammatory mediators, of which IL-10 plays a central overarching role. This represents an exciting avenue of research that will hopefully usher unique immunomodulatory therapies, changing the management of patients with acute neurologic injury from supportive measures to active therapeutic care to improve patient outcomes.

## Author Contributions

JG and SS drafted the manuscript. JL and SD made substantial contributions to the conception and design of the work and wrote the manuscript. JL, HP, NE, SR, BH, SB, and SD revised the manuscript. All authors have approved the manuscript for publication.

## Conflict of Interest Statement

The authors declare that the research was conducted in the absence of any commercial or financial relationships that could be construed as a potential conflict of interest.
